# Supplementary material for: Crystal structures of MHC class I complexes reveal the elusive intermediate conformations explored during peptide editing
Source: Nat Commun. 2023 Aug 18;14:5020. doi: 10.1038/s41467-023-40736-6 (PMC10439229; doi:10.1038/s41467-023-40736-6)
Supplement: Supplementary file 1 — Supplementary Information [file 41467_2023_40736_MOESM1_ESM.pdf]

Crystal structures of MHC class I complexes reveal the elusive intermediate conformations explored during peptide editing

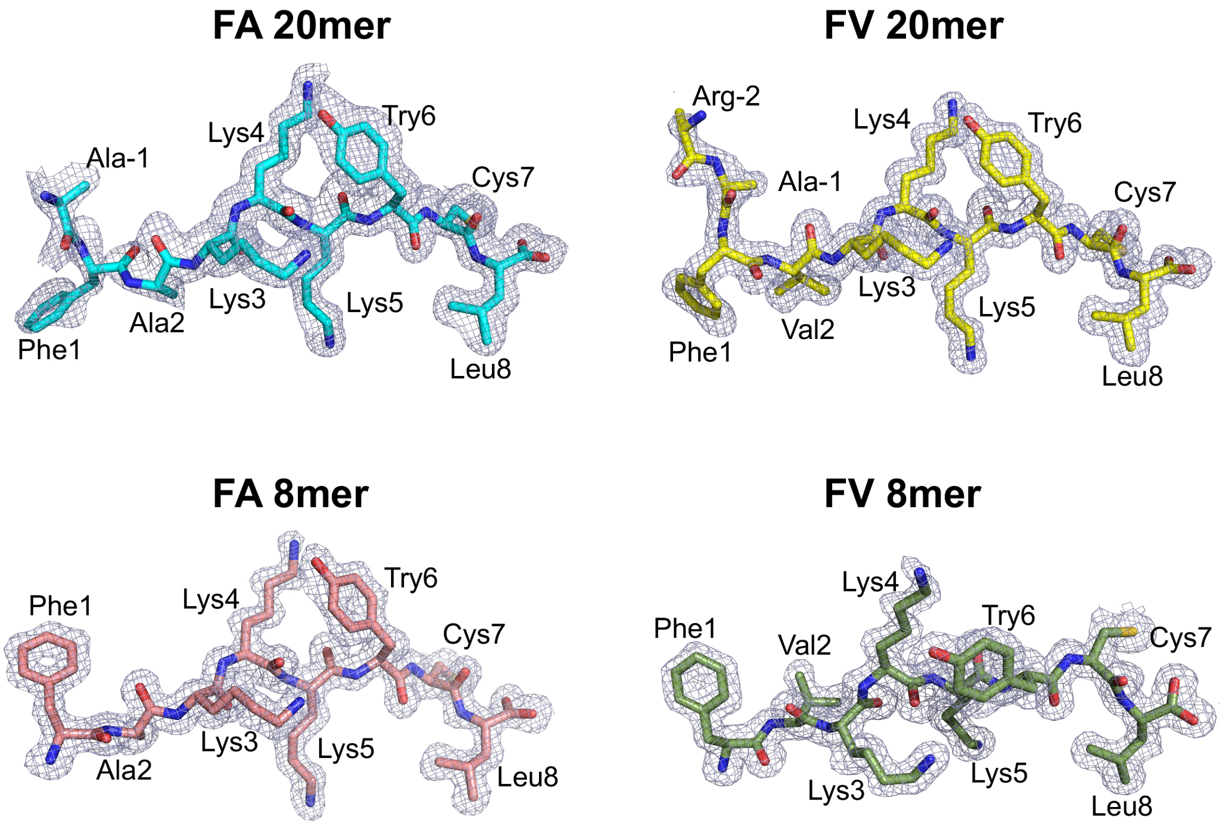

Supplementary Figure 1. Electron density maps of bound peptides. The 2mFo-DFc electron density map, contoured at 1σ level, is shown as mesh around bound FA 20mer (cyan), FV 20mer (yellow), FA 8mer (pink), and FV 8mer (green) peptides. The density is clearly visible for all residues, including the backbone and methyl side chain of P-1 Ala (20mers) and the backbone and part of the aliphatic side chain of P-2 Arg (FV 20mer).

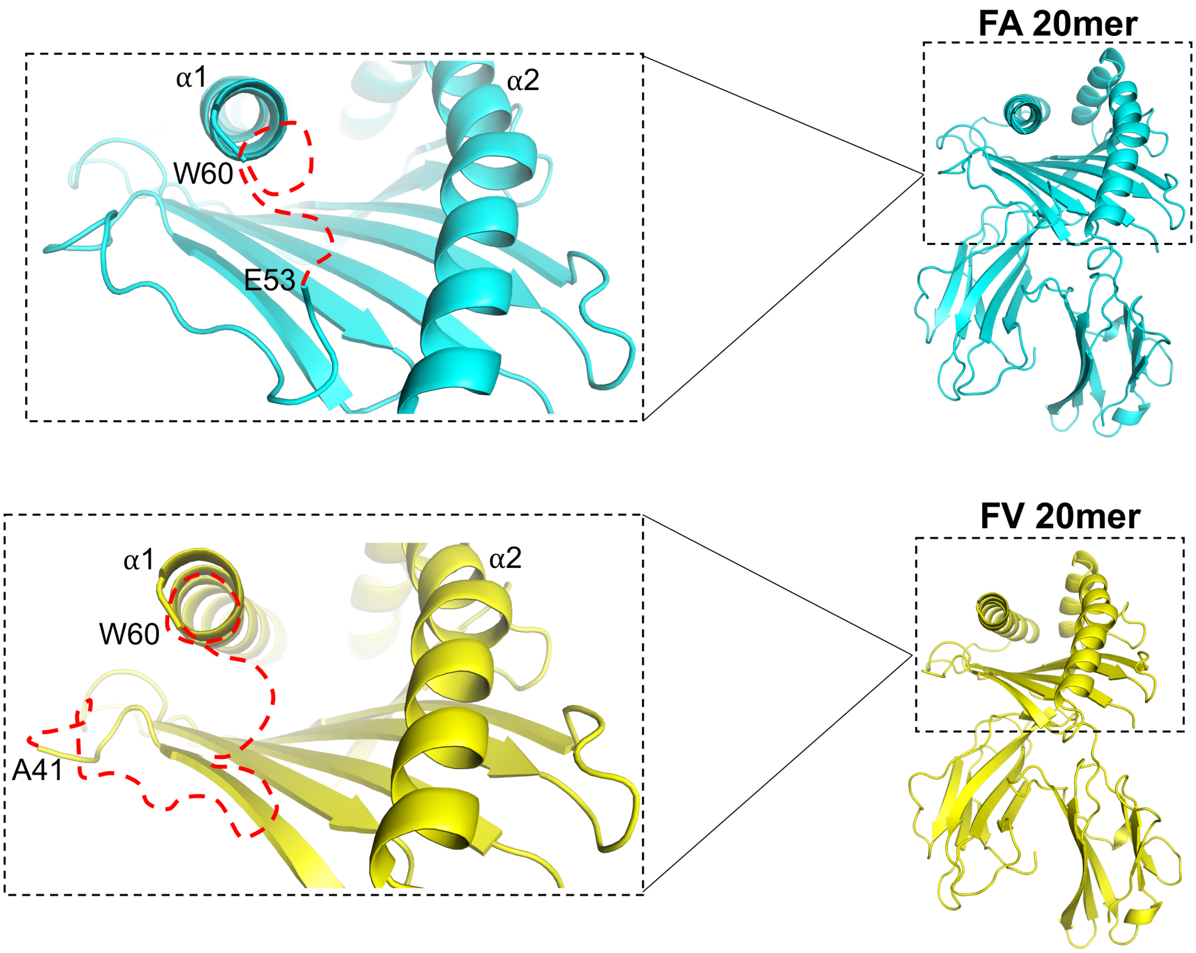

Supplementary Figure 2. Open-ended nature of MHC I groove. The widely open-ended nature of HLA-B8E76C groove in FA and FV 20mer structures with disordered residues Gln54 to Tyr59 and Ser42 to Tyr59, respectively, shown as red dashed lines.

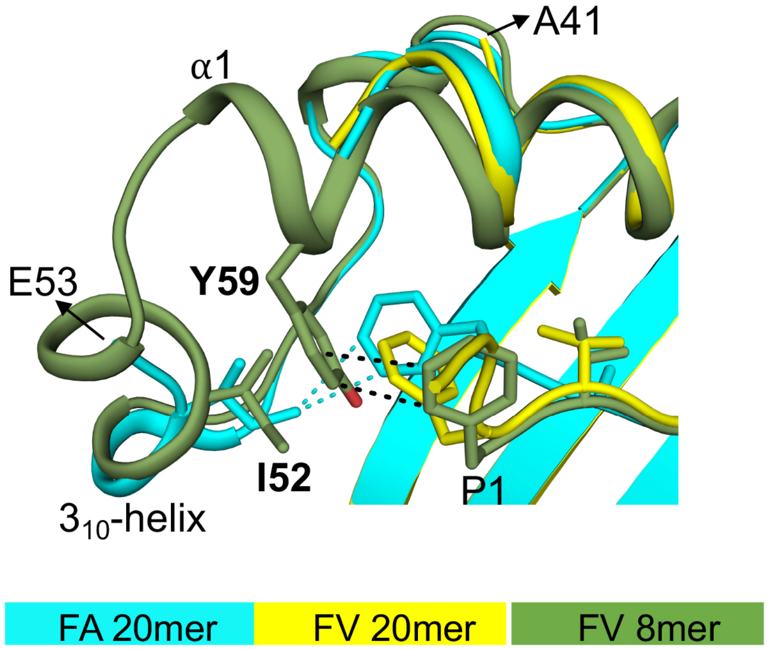

Supplementary Figure 3. Roles of MHC I residues Tyr59 and Ile152 at the N-terminus of the groove. Superimposition of FA and FV 20mer structures with FV 8mer structure. The P1 Phe side chain of FV 8mer occupies its canonical position and interacts with Tyr59, while the bulky P1 Phe side chains of FA and FV 20mers clash with Tyr59 causing conformational disorders in residues Gln54 to Tyr59. The P1 Phe side chain of FV 8mer has no interaction with Ile52.

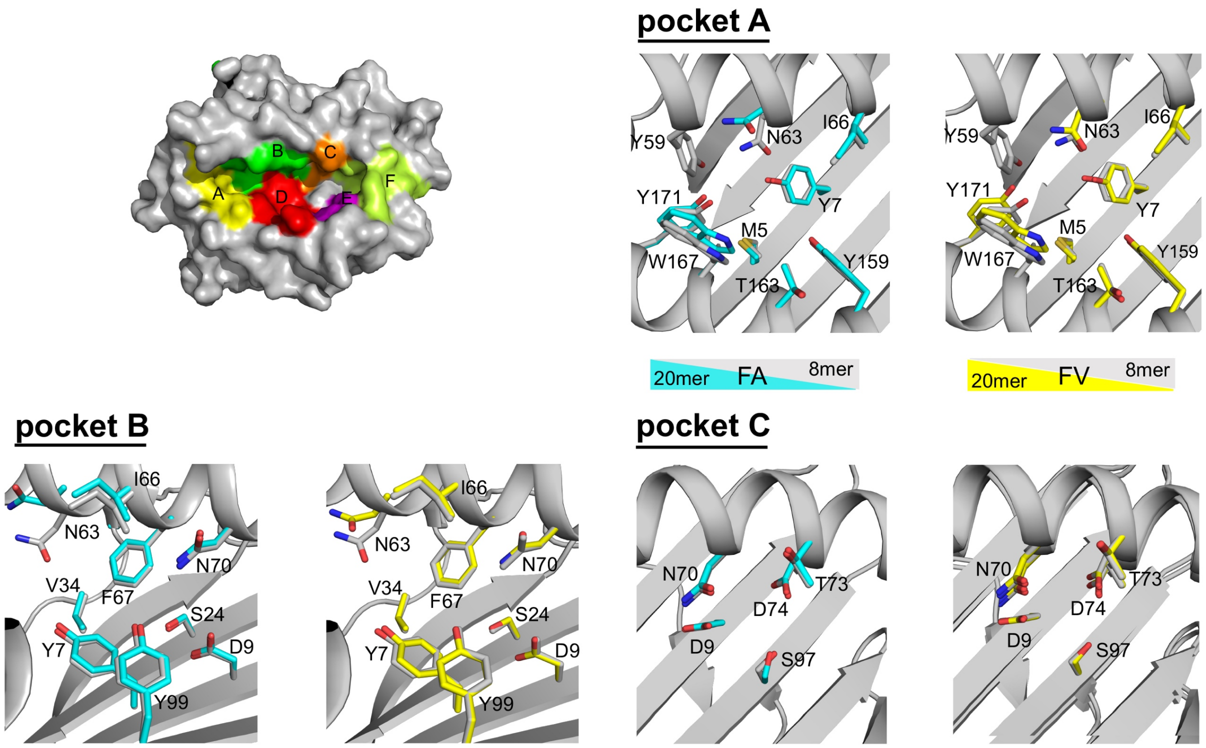

Supplementary Figure 4. Binding pockets along HLA-B8E76C groove. Molecular surface of HLA-B8E76C groove showing pockets A to F. Superimposition of FA 20mer and 8mer structures and of FV 20mer and 8mer structures showing the side chain orientations of MHC I residues in pockets A, B, and C. Pocket A, Tyr59 and Asn63 are the most divergent residues in both FA and FV 20/8mer structures, with some differences also seen in Tyr171 and Trp167. Pocket B, Asn63 (at the boundary of pockets A and B) is the most divergent residue in both FA and FV 20/8mer structures. No changes in MHC I side chain orientations were observed in pocket C.

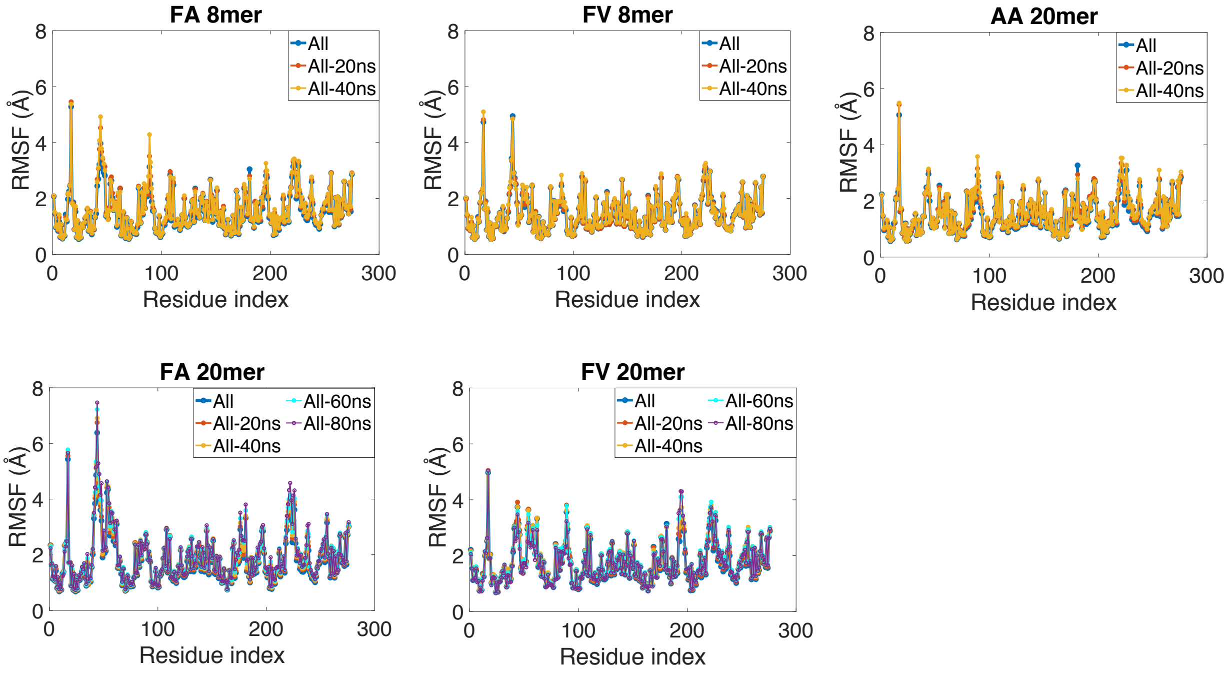

Supplementary Figure 5. Validation on the equilibrations of MD simulations by time-course analysis on RMSF curves. For each system, the root mean square fluctuation (RMSF) curves were obtained from the ensembles with different cut-off of the initial parts of the simulation trajectory, where "All" means that the whole trajectory was used, while "All-xxns" means that the RMSF was calculated from the ensemble with the first xx ns of the trajectory discarded. The RMSF curves did not change with different cut-offs of the initial parts of the simulation trajectories, indicating that the systems are well equilibrated.

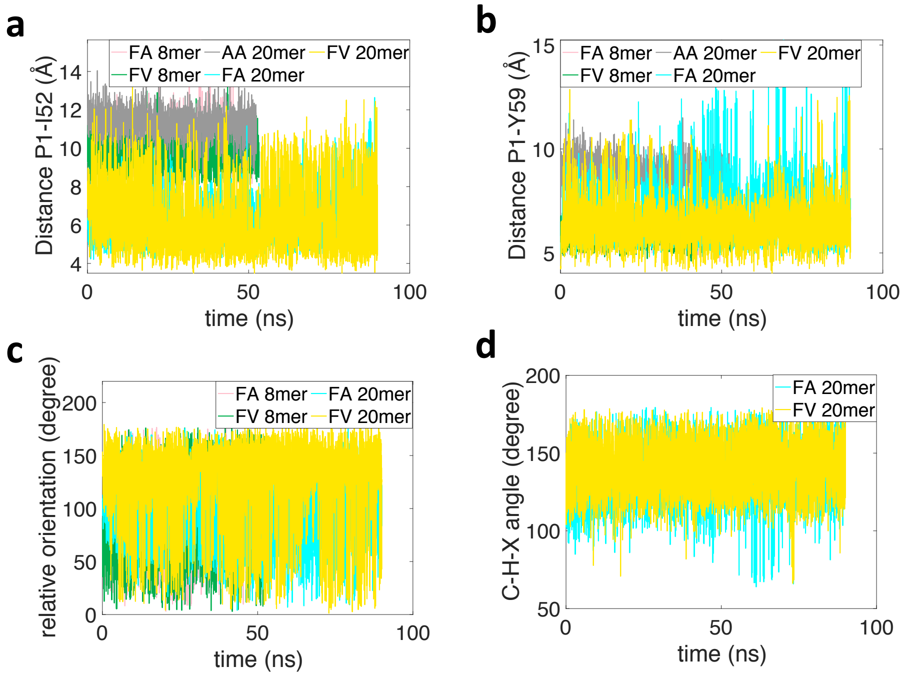

Supplementary Figure 6. Validation on the equilibrations of MD simulations by time-course analysis on different geometry parameters. The time traces of different geometry parameters were drawn for the replica MD simulations at 310 K. The measured geometry parameters include: a, the inter-residue distance between peptide P1 and Ile52. b, the inter-residue distance between peptide P1 and Tyr59. c, the relative orientation between the aromatic rings of P1 and Y59. d, the angle C-H-X (X being the center of P1 Phe aromatic ring) defined as the bond angle formed by atoms Cδ of Ile52, the hydrogen atom covalently bonded to Cδ, and the geometrical center of Phe aromatic ring. We can see that all the geometrical parameters fluctuate back and forth in a wide range, indicating that the simulations have established ergodicity and reached thermal equilibration.

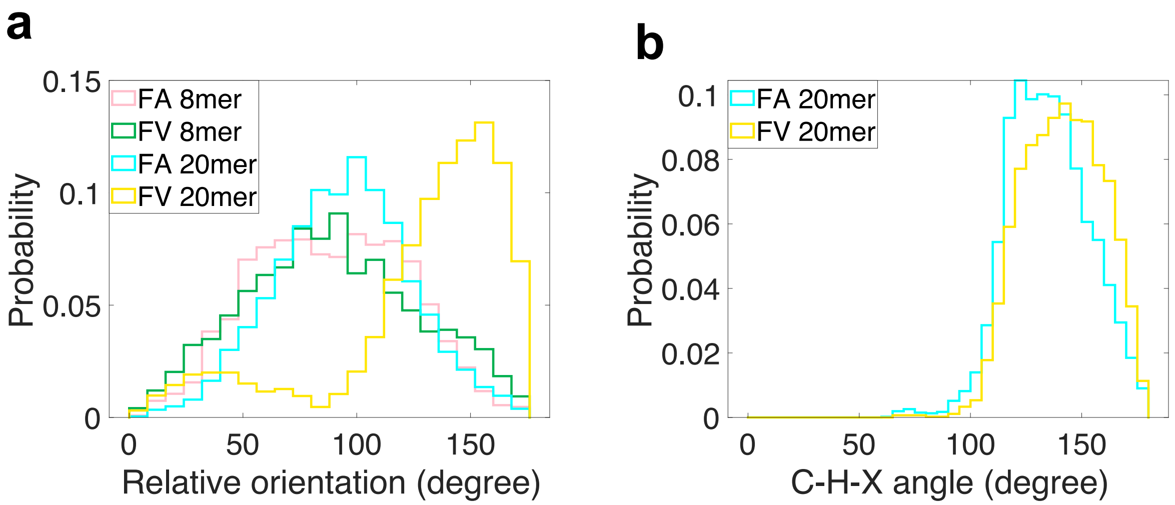

Supplementary Figure 7. Geometrical parameters of interaction between peptide P1 residue and N-terminal MHC I residues Tyr59 and Ile52. a, Probability distributions of relative orientations between the aromatic rings of P1 Phe and Tyr59 in FA and FV 8/20mer complexes (see text). b, Probability distributions of angle C-H-X (X being the center of P1 Phe aromatic ring) for FA and FV 20mers showing that the angles are mostly in the range 120° to 150°. The angle C-H-X was defined as the bond angle formed by atoms Cδ of Ile52, the hydrogen atom covalently bonded to Cδ, and the geometrical center of Phe aromatic ring.

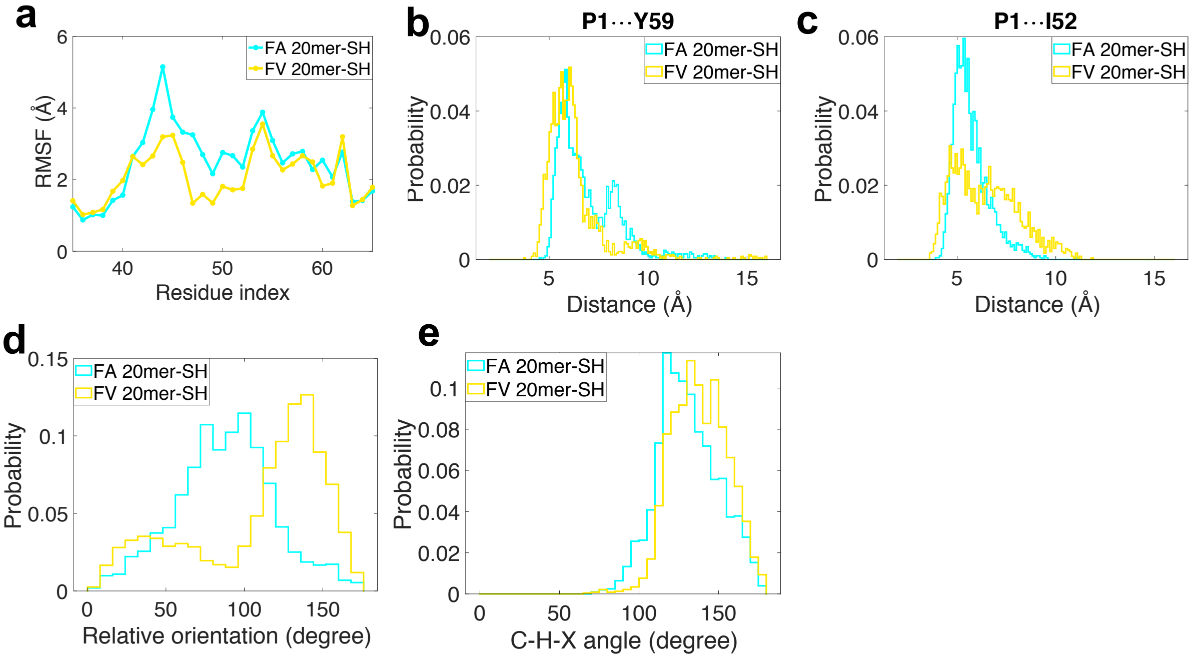

Supplementary Figure 8. Conformational flexibility and probability distributions of geometrical parameters of non-disulfide bonded HLA-B8E76C FA and FV 20mer complexes. a, Root mean square fluctuation (RMSF) values of individual residues along the heavy chain of HLA-B8E76C loaded with FA and FV 20mers. Two distinct regions are highlighted, residues 41 to 46 (peptide-independent) and residues 52 to 62 (peptide-dependent). b, Probability distributions of inter-residue distance between peptide P1 and Tyr59 (P1···Y59) and c, between peptide P1 and Ile52 (P1···YI52). d, Probability distributions of relative orientations between the aromatic rings of P1 Phe and Tyr59. e, Probability distributions of angle C-H-X (X being the center of P1 Phe aromatic ring). The angle C-H-X is defined as the bond angle formed by atoms Cδ of Ile52, the hydrogen atom covalently bonded to Cδ, and the geometrical center of Phe aromatic ring.

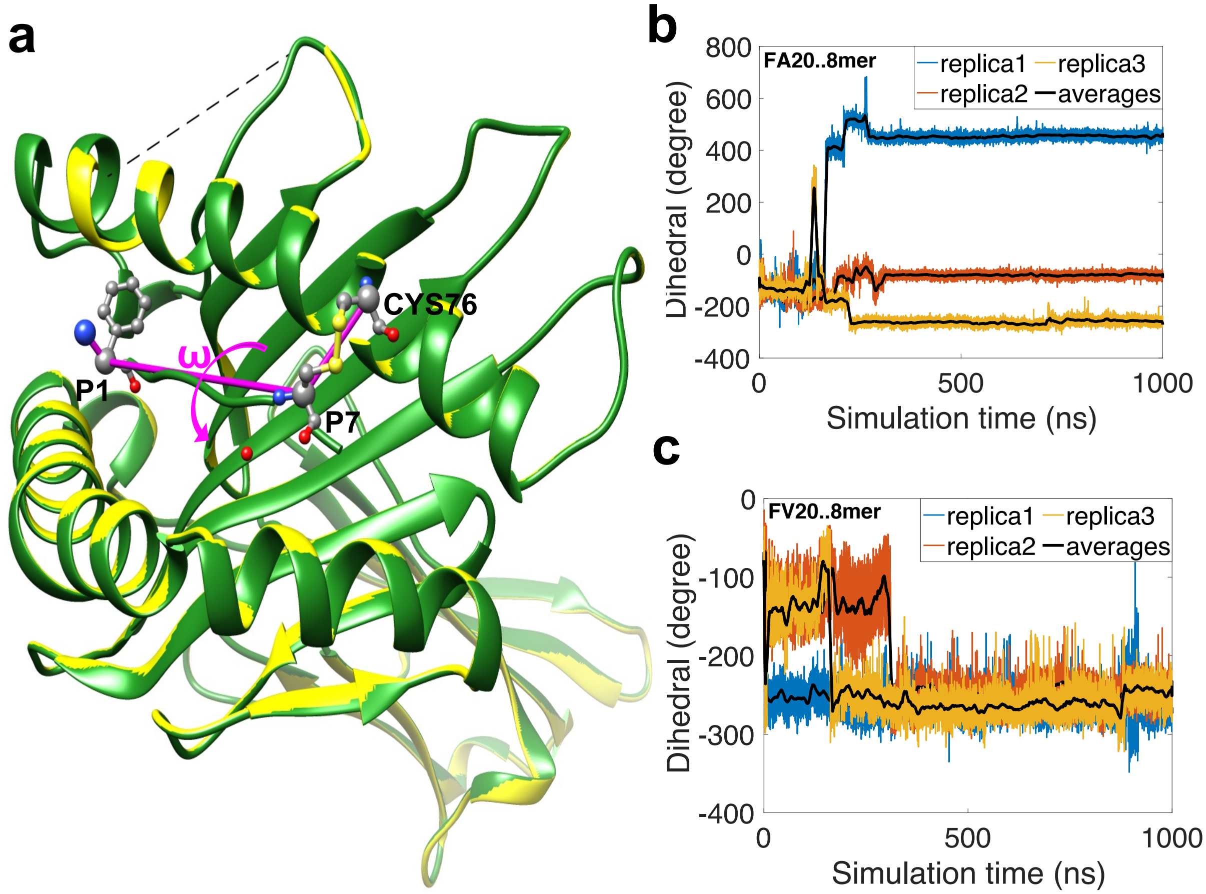

Supplementary Figure 9. Rotation of peptide terminal amino group in the A pocket for FA and FV 20mer complexes. a, The dihedral angle ω used for analysis of rotations of peptide N-terminal amino groups is defined by four atoms, Cys76:CA-P7:CA-P1:CA-P1:N, as exemplified with FV20..8mer (yellow) (see main text). The FV 8mer structure is also shown in green. The dihedral angle ω is labelled in magenta. b, c, Time traces of the dihedral angle ω for FA20..8mer and FV20..8mer, respectively, in three replicate MD simulations. The black lines represent values of ω with 10 ns running average. For replica 1 of FA20..8mer and FV20..8mer, the initial coordinate and simulation input files, and coordinate files of the final output, are provided in Supplementary Data (Supplementary Data 1 contains the input and output files, while Supplementary Data 2 contains the parameter files).

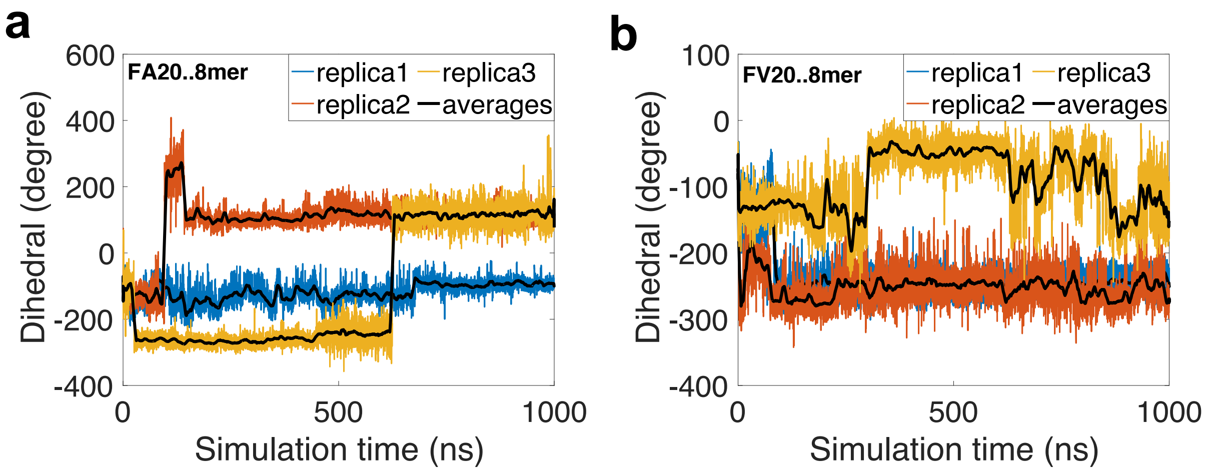

Supplementary Figure 10. Rotation of peptide terminal amino group in the A pocket for non-disulfide bonded FA and FV 20mer complexes. a, b, Time traces of the dihedral angle ω, as defined in Supplementary Fig. 9a, for non-disulfide bonded FA20..8mer and FV20..8mer, respectively, in three replicate MD simulations. The black lines are values of ω with 10 ns running averages. For both non-disulfide bonded FA and FV 20mer complexes, two out of three replicas (orange/yellow and blue/orange, respectively) stabilized at the canonical position of peptide N-terminal amino group within 1 μs. For replica 2 of FA20..8mer and FV20..8mer, the initial coordinate and simulation input files, and coordinate files of the final output, are provided in Supplementary Data (Supplementary Data 1 contains the input and output files, while Supplementary Data 2 contains the parameter files).

Supplementary Table 1. Data collection and refinement statistics

	FA8mer	FA20mer	FV8mer	FV20mer
**Data collection** *†				
Space group	P212121	P212121	P212121	P212121
Cell dimensions	50.58, 81.27,	50.74, 81.39,	50.65, 81.23,	50.76, 81.15,
a, *b*, *c* (Å)	110.45	110.99	110.18	110.58
α, β, γ (°)	90	90	90	90
Resolution (Å)	65.47-1.37 (1.42-1.37)	32.82-1.13 (1.16-1.13)	19.61-1.49 (1.51-1.49)	24.00-1.22 (1.25-1.22)
R_{merge}	0.068	0.084	0.099	0.072
$I / \sigma I$	29.1 (2.1)	20.0 (3.6)	13.5(2.9)	10.6(3.1)
Completeness (%)	99.50 (97.27)	85.11 (25.30)	96.49 (83.93)	98.12 (94.50)
Redundancy	4.2(3.5)	1.7(1.0)	8.2(6.6)	9.1(8.1)
**Refinement**				
Resolution (Å)	65.47-1.37 (1.42-1.37)	32.82-1.13 (1.16-1.13)	19.61-1.49 (1.51-1.49)	24.00-1.22 (1.25-1.22)
No. reflections	95762	137770	72099	126660
R_{work} / R_{free}	18.1/22.4 (23.6/26.8)	18.9/21.7 (41.0/48.7)	17.9/21.1 (31.0/36.2)	19.9/21.2 (26.2/27.5)
No. atoms				
Protein	3148	3103	3150	2999
Water	247	379	384	357
B-factors				
Protein	19.6	34.84	19.3	19.9
Water	23.8	41.7	28.0	28.8
R.m.s. deviations				
Bond lengths (Å)	0.018	0.018	0.016	0.018
Bond angles (°)	2.19	1.61	1.61	1.99
PDB code	8E13	8E2Z	8E8I	8EC5

*One crystal was used for each dataset. †Values in parentheses are for highest-resolution shell.

Supplementary Table 2. MD simulations setup

MD systems	Box dimensions (octahedron, nm)			Total number of atoms	Total number of water molecules	Salt concentration (M)
	XX	YY	ZZ			
	XY	XZ	YX			
	YZ	ZX	ZY			
FA 8mer	10.01399	9.44128	8.17639	77825	23868	0.1
	0.00000	0.00000	3.33800			
	0.00000	-3.33800	4.72064			
FV 8mer	9.99391	9.42235	8.15999	77,810	23,861	0.1
	0.00000	0.00000	3.33130			
	0.00000	-3.33130	4.71117			
AA 20mer	10.60108	9.99478	8.65574	92,446	28,673	0.1
	0.00000	0.00000	3.53370			
	0.00000	-3.53370	4.99740			
FA 20mer	10.54710	9.94390	8.61167	92,058	28,535	0.1
	0.00000	0.00000	3.51569			
	0.00000	3.51569	4.97195			
FV 20mer	11.08829	10.45415	9.05356	105,489	33,011	0.1
	0.00000	0.00000	3.69609			
	0.00000	-3.69609	5.22708			
FA 20..8mer	10.13997	9.56006	8.27925	78,786	24,183	0.1
	0.00000	0.00000	3.37999			
	0.00000	-3.37999	4.78003			
FV 20..8mer	10.11679	9.53820	8.26032	78,049	23,941	0.1
	0.00000	0.00000	3.37226			
	0.00000	-3.37226	4.76910			
