# Supplementary material for: Crystal structures of MHC class I complexes reveal the elusive intermediate conformations explored during peptide editing
Source: Nat Commun. 2023 Aug 18;14:5020. doi: 10.1038/s41467-023-40736-6 (PMC10439229; doi:10.1038/s41467-023-40736-6)
Supplement: Supplementary file 3 — Description of Additional Supplementary Files [file 41467_2023_40736_MOESM3_ESM.pdf]

Description of Additional Supplementary Files

File Name: Supplementary Data 1

Description: FigS9b_input_replica1: The input coordinate file for the MD simulation replica 1 on the rotation of peptide terminal amino group in the A pocket of FA20..8mer.

FigS9b_output_replica1: The final output coordinate file for the MD simulation replica 1 on the rotation of peptide terminal amino group in the A pocket of FA20..8mer.

FigS9c_input_replica1: The input coordinate file for the MD simulation replica 1 on the rotation of peptide terminal amino group in the A pocket of FV20..8mer.

FigS9c_output_replica1: The final output coordinate file for the MD simulation replica 1 on the rotation of peptide terminal amino group in the A pocket of FV20..8mer.

FigS10a_input_replica2: The input coordinate file for the MD simulation replica 2 on the rotation of peptide terminal amino group in the A pocket of non-disulfide bonded FA20..8mer. FigS10a_output_replica2: The final output coordinate file for the MD simulation replica 2 on the rotation of peptide terminal amino group in the A pocket of non-disulfide bonded FA20..8mer. FigS10b_input_replica2: The input coordinate file for the MD simulation replica 2 on the rotation of peptide terminal amino group in the A pocket of non-disulfide bonded FV20..8mer. FigS10b_output_replica2: The final output coordinate file for the MD simulation replica 2 on the rotation of peptide terminal amino group in the A pocket of non-disulfide bonded FV20..8mer.

File Name: Supplementary Data 2

Description: minim: The parameter file for the energy minimization stage in MD simulations on all the systems. nvt: The parameter file for the NVT thermal equilibration stage in MD

simulations on all the systems. npt: The parameter file for the NPT thermal equilibration stage in MD simulations on all the systems. md: The parameter file for the production run in MD simulations on all the systems.
